# Numerical and Experimental Study of Colored Magnetic Particle Mapping via Magnetoelectric Sensors

**DOI:** 10.3390/nano13020347

**Published:** 2023-01-14

**Authors:** Ron-Marco Friedrich, Mohammad Sadeghi, Franz Faupel

**Affiliations:** Chair for Multicomponent Materials, Institute of Materials Science, Kiel University, Kaiserstr. 2, D-24143 Kiel, Germany

**Keywords:** magnetic particle mapping, nanoparticle, magnetoelectric, inverse optimization, projected gradient descent

## Abstract

Colored imaging of magnetic nanoparticles (MNP) is a promising noninvasive method for medical applications such as therapy and diagnosis. This study investigates the capability of the magnetoelectric sensor and projected gradient descent (PGD) algorithm for colored particle detection. In the first step, the required circumstances for image reconstruction are studied via a simulation approach for different signal-to-noise ratios (SNR). The spatial accuracy of the reconstructed image is evaluated based on the correlation coefficient (CC) factor. The inverse problem is solved using the PGD method, which is adapted according to a nonnegativity constraint in the complex domain. The MNP characterizations are assessed through a magnetic particle spectrometer (MPS) for different types. In the experimental investigation, the real and imaginary parts of the MNP’s response are used to detect the spatial distribution and particle type, respectively. The experimental results indicate that the average phase difference for CT100 and ARA100 particles is 14 degrees, which is consistent with the MPS results and could satisfy the system requirements for colored imaging. The experimental evaluation showed that the magnetoelectric sensor and the proposed approach could be potential candidates for color bio-imaging applications.

## 1. Introduction

Magnetic resonance imaging (MRI), computed tomography (CT), and single-photon emission computed tomography (SPECT) have been widely used in disease therapy and for clinical purposes as medical imaging modalities [1,2,3]. Nevertheless, using X-rays or a strong magnetic field might pose some issues, such as causing health-related problems, making the system complicated, and being costly [2,3].

Magnetic particle imaging (MPI), which was first introduced in 2005 [4], has recently gained popularity in measuring the spatial distribution of magnetic nanoparticles (MNPs). However, compared to standard clinical imaging, the image resolution needs to be improved [5]. In the MPI device, a system matrix should be developed to reconstruct the image for the spatial distribution of MNPs. Two well-known approaches for creating a system matrix are model-based [6] and measurement-based [7]. In the measurement-based method, the drive field moves through the sample area and acts as a sensitive spot to increase the spatial resolution. The field-free point (FFP) [8] and the field-free line (FFL) [9] are two common methods for scanning procedures. The final images can then be reconstructed using, e.g., a filtered back-projection algorithm [10].

In the model-based reconstruction approach, a precise field simulator that is based on the Biot–Savart law needs to be developed. Although, the image quality of the model-based approaches is promising, creating a model is challenging due to the complex dynamic behavior of the MNPs [9]. An iterative numerical algorithm is required in this method to solve the linear system equations and reconstruct the final image. The most utilized optimization algorithms for MPI are the simultaneous algebraic reconstruction technique (SART) [11], iterative conjugate normal residual (CGNR) [12], subspace Barzilai–Borwein nonnegative least-squares (SBB-NNLS) [13] and fast iterative shrinkage thresholding (FISTA) [14]. Several studies have reported the feasibility of a pickup coil as a sensor unit for MPI applications [9,15,16]. Magnetoelectric composites have extensive applications as mechanical actuators [17] and magnetic sensors [18,19] due to high sensitivity and a reasonable limit of detection for medical applications [20]. A recently developed MPM approach has been introduced by utilizing the ME sensor as a detection unit [14,21].

For many biological applications, the ability to detect particles from different sources and environments would be highly desirable [22,23]. The imaging of distinct particle responses adds a promising layer to the foundation of magnetic particle imaging, known as Multi-colored MPI. Nanoparticle properties, such as core material, diameter, magnetization response, and anisotropy, affect relaxation behavior. As a result, they can serve as potential parameters for particle type detection [11]. In this regard, the phase response of MNPs has been used for colored MPI detection systems [16]. The magnetic response of the MNPs depends on environmental properties such as temperature and viscosity. This concept opens another path for MPI to differentiate particles according to their type and environmental temperature. Temperature monitoring of magnetic nanoparticles has been numerically [12] and experimentally [11,24,25,26] evaluated successfully in the MPI research field. Experimental investigation indicates a trade-off between temperature and spatial resolution, as shown for a scanning magnetic particle spectrometer imaging system [11]. Moreover, unknown particle characteristics for different temperature ranges could be a practical limitation [24,26].

The conventional MPI system needs large DC gradient fields to produce FFL or FFP. Generating a sufficient signal in the pick-up coils at the kHz regime needs huge power supplies, which makes the system complicated and expensive. Furthermore, the large DC gradient fields will apply a significant force on the cells/MNPs. The heat dissipation in coils is another source of challenges in these systems. Although many efforts have been made to develop the noninvasive MPI method, designing a color imaging system with a reliable particle type distinction is still a challenging and urgent research topic. In this paper, a novel colored magnetic particle mapping approach has been introduced with the aim of implementing a cheap, small, and sensitive magnetoelectric sensor and phase detection technique that requires neither strong magnetic field gradients nor high-power units. The reconstruction requirement and image prediction were investigated via a simulation study for different signal-to-noise ratios. The characteristics of MNPs, such as amplitude, phase, and harmonic response, were assessed via magnetic particle spectrometry (MPS). A fabricated thin film magnetoelectric composite was used as a sensor, and a bio-imaging apparatus was developed for experimental evaluation. The forward model was established based on the system matrix considering the sensor sensitivity axis and dipole direction. The spatial distribution of two particle types was investigated for the third harmonic response. The scanning area was discretized to the equidistant Cartesian grid, and the inverse problem was solved based on the projected gradient descent (PGD) algorithm. In this proof-of-concept study, the simulation and experimental results confirmed the ability of the proposed approach for colored imaging of magnetic nanoparticles.

## 2. Material and Methods

### 2.1. Experimental Setup

**Figure 1:**
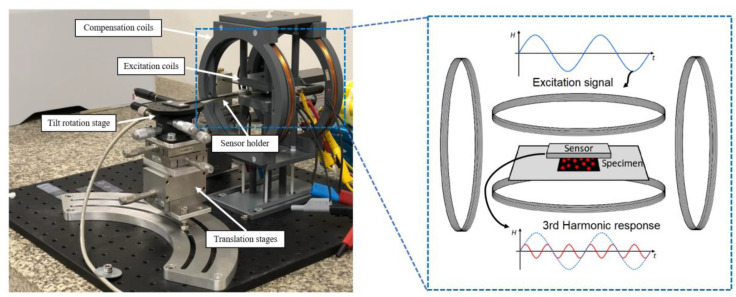
The magnetic particle imaging apparatus; the left side shows the fabricated setup and the right side depict a schematic diagram of the core.

The structure of the magnetic particle mapping (MPM) setup, including the sensor, magnetic field source, electric appliances, and the MPM controller software, has been illustrated in Figure 1. An exchange-biased magnetoelectric sensor was employed as a detection unit. The sensor was fabricated in the form of a cantilever having a 3 mm length and 1 mm width, which is made of 50 μm poly-silicon consisting of both the piezoelectric and magnetostrictive phases on top. This resonant cantilever-type structure enables the use of increased sensitivities and detection limits at the resonance frequency. A stack of the highly magnetostrictive alloy Fe_70.2_Co_7.8_Si_12_B_10_ (FeCoSiB) is sputtered for about 1 μm [18]. The highly piezoelectric aluminum nitride (2 μm-AIN) is also sputtered by a low-temperature deposition process. The sensor is released using wet/dry etching technologies. In order to use the exchange bias effect, the sensor was annealed (30 min at 250 °C) under a 1 kG magnetic field that was employed 60° to the long axis of the cantilever. In this way, the highest sensitivity occurs at zero external magnetic fields. More details about the sensor fabrication procedure and exchange bias effect can be found in our previous publication [18].

The excitation AC magnetic field was generated with a pair of Helmholtz coils. Another coil arrangement was utilized to apply a low-amplitude AC field and compensate for an unwanted background signal in the sensing area due to nonlinear excitation at the sensor’s resonance frequency. The essential power was supplied using the signal generator (RME Fireface UC) and an AC amplifier (PAS2002 audio AMP). For enhancing the signal-to-noise ratio (SNR), the sensor output response was amplified by a low-noise charge amplifier (CA). More details about the amplifier design and noise analysis can be found in our previous publication [27]. The sample positioning was controlled automatically via 2D XY-stage and linear step motors. Meanwhile, the sensor holder could be adjusted manually with different degrees of freedom, including tilt, rotation, and translation with a precise micrometer head. A schematic diagram of the electronic appliances has been demonstrated in Supplementary Data, Figure S1. As can be seen, all elements were controlled by the developed MPM controller, which was hosted on a computer with MATLAB software. In addition, data analysis was performed via the developed GUI evaluator program.

### 2.2. Sample Preparation

**Figure 2:**
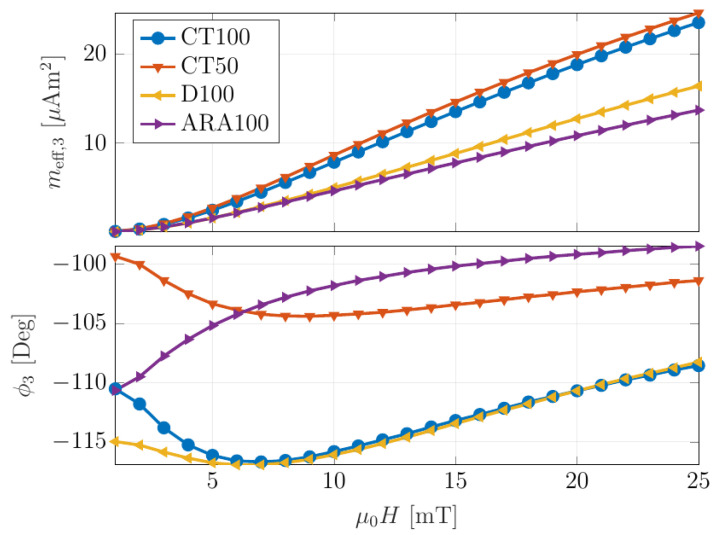
Effective magnetic moments’ response of the 3rd harmonic for 150 μL of different MNPs type.

This study uses the phase difference in the reconstruction procedure to distinguish between particle types. In this regard, differentiation in the phase of particle response is necessary. Magnetic particle spectrometry (MPS) was performed on the different MNP types to evaluate the phase and amplitude of particle response at 25 mg/mL concentration. MPS spectra were measured at 2.5 kHz for 10 mT, aligning with the readout scheme. The different types of superparamagnetic nanoparticles (magnetite core in citric acid matrix) that are specially designed for the MRI diagnostic were purchased from chemicell GmbH (Berlin, Germany). In the measurement scheme, the MNPs were excited with an alternating magnetic field at 2.5 kHz, roughly one-third of the resonance frequency of the ME sensor. Consequently, the third harmonic of the nanoparticle response at 7.5 kHz was measured. Figure 2 shows the effective magnetic moment response of the 3rd harmonic for different nanoparticles, including CT 50, CT 100, D100, and ARA 100. The MPS spectra can be found in Figure S2 (Supplementary Data).

The CT100 and ARA100 particles were selected due to their large amplitudes and phase differences at the chosen magnetic excitation (10 mT). The selected MNPs were placed in distinct containers in a cylindrical sample holder (Figure S3 of Supplementary Data) of 4 mm thickness. The nanoparticles were filled in the holder’s outer volumes, opposite each other, with 14 μL for each type. To prevent evaporation, adhesive tape was used to seal the containers.

### 2.3. Image Reconstruction

The forward modeling approach is introduced in this section, and then an algorithm for the color imaging reconstruction procedure is presented.

In the mapping process, the model matrix mathematically describes the relationship of particle distribution to the measured signal. To distinguish different particle types, one can incorporate the amplitude and phase responses of nanoparticles into the model matrix. For this, the model matrix can be scaled via the particle amplitude and phase response (complex factor). In the reconstruction, the particle behavior can also be extracted by using only a single-model matrix for a single particle type if the underlying model matrices only differ in a complex factor in terms of amplitude and phase. In MPM, this is the case and we, thus, proceed with this approach, as will be outlined further below.

The magnetic field ($B_{m}$) for the measured positions ($r_{m}$) can be calculated by the Fredholm integral equation:

(1) $$\int_{\Omega }^{}\rho \left(r\right)B_{D}\left(r_{m},r\right)d^{3}r=B_{m}\left(r_{m}\right)$$

where ρ is a spatial magnetic particle distribution, $B_{D}$ is projected dipole field, and Ω is the domain in which particles are present. The mentioned integral could be discretized to a linear equations’ system as expressed in Equation (2).

(2) Ax=b

where b is the superposition of nanoparticles’ magnetic fields’ response for different measurement positions, x is the spatial MNP distribution, and A indicates the model matrix. The model matrix includes the magnetic dipoles’ orientation and the sensitivity axis of the sensor. More detail about the model could be found in our previous publication [28].

The particle distribution was computed based on a superposition of the particle responses for $p_{1}$ and $p_{2}$ MNP types using Equations (3)–(5) where $x_{i}$ is an entry in the particle distribution vector.

(3) $$x_{i}=x_{i,p_{1}}+x_{i,p_{2}}$$

(4) (Re(xi)Im(xi))=(|a1|cos(φ1)|a2|cos(φ2)|a1|sin(φ1)|a2|sin(φ2))(|xi,p1||xi,p2|)

(5) (Re(xi)Im(xi))=Q(|xi,p1||xi,p2|)

Here, the Matrix Q incorporates the different particle behaviors via their amplitudes $a_{i}$ and phases $\varphi_{i}$. Additionally, given the real and imaginary part of the reconstruction vector, one can compute the corresponding particle concentrations via the mapping $Q^{-1}$. This already necessitates that for colored imaging there must be a phase difference between the particles, as otherwise the determinant would be zero. However, since negative particle amounts are not physically meaningful, a nonnegativity constraint needs to be considered in the reconstruction procedure. The mentioned constraint, for the solution domain with an absolute real value response, means that the solution lies in the nonnegative orthant. The real and imaginary parts of the particle response for a complex domain must lie on a line in the complex plane. To this end, the entries $x_{i}$ can be assumed to lie in a polyhedral cone, spanned by the complex lines stemming from the different particle behaviors (Figure S4 of Supplementary Data).

To enforce this as a constraint in the reconstruction, a projection operator (Snippet S1 of Supplementary Data) for the cone can be used in conjunction with the projected gradient descent method [28]. The projection operator $P_{+}^{Q}$ for the cone can be realized by projection into the corresponding half spaces created by the lines in the complex plane and an additional nonnegativity constraint to only include nonnegative scalars.

According to Equation (6), the PGD method was applied with the nonnegativity projection constraint.

(6) $$x_{k+1}=P_{+}^{Q}\left(x_{k}-\alpha A^{T}\left(Ax_{k}-b\right)\right)$$

where $x_{k}$ is the solution of the system matrix for *k*th iteration, α is the step size, and $P_{+}^{Q}$ is the projection operator. The proposed iterative optimization method for colored MPM can be written as shown in Algorithm 1. Here, x denotes the complex spatial particle distribution, whereas y denotes the spatial particle distributions of the individual particles yielded by the application of the mapping $Q^{-1}$.
**Algorithm 1.** Adopted PGD method for colored MPM application.1:given: iterations K, data b, estimate of noise standard deviation δ, stopping condition for discrepancy principle η, particle type matrix Q, cone projection operator for particle types $P_{+}^{Q}$.2:initialize forward operator A, particle distribution x.3:for k = 1 to K do.4:$\left[x_{k+1},y_{k+1}\right]=P_{+}^{Q}\left(x_{k}+{\left\|A\right\|}_{2}^{-2}A^{T}\left(b-Ax_{k}\right)\right)$5:*if*$\frac{\|Ax_{k+1}-{b\|}_{2}}{\delta }\leq \eta \;then$6:*return*$y_{k+1}$7:*end if*8:*end for*9:*return*$y_{k+1}$

In the reconstruction process, a 2D Cartesian plane was considered based on the measurement positions with the same dimensions and difference in height (2.5 mm). For a 3D sample, height does not affect the phase responses of MPNs. Hence, a 2D model was used for a 3D sample as a proof of work.

### 2.4. Simulation Procedure

**Figure 3:**
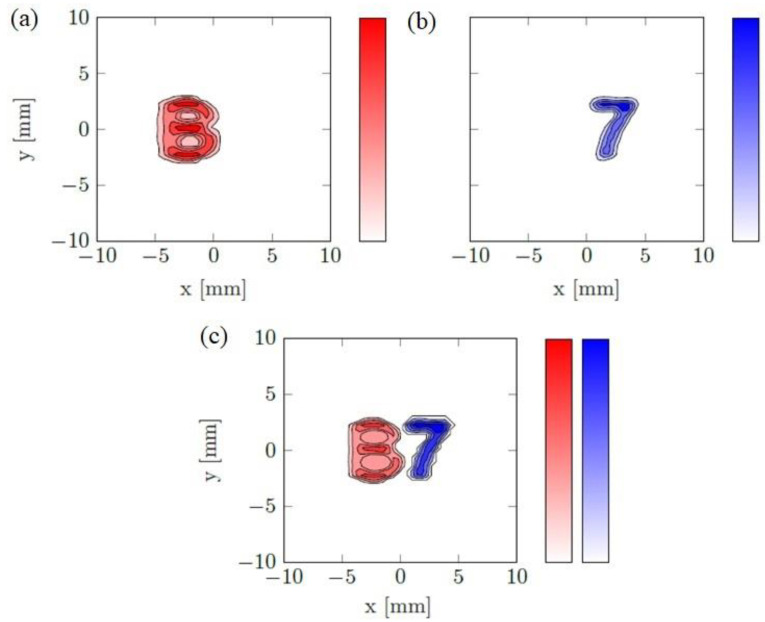
The simulation results for the reconstruction of two different MNP types; (**a**) Red and (**b**) blue color indicate MNP type I and II; (**c**) Combined reconstructed image. The color bar indicates the particle amount.

Simulation studies were performed based on a 2D planar system to assess the essential circumstances for the colored reconstruction procedure. The letters “B7” were used to constitute a spatial particle distribution (seen further below in Figure 3). Each letter has a specific phase corresponding to the particle’s response. As a numerical assumption, the amplitude per unit mass for both particles was set to the same value. The source domain was selected based on the exact dimensions of the measurement domain, which includes 53 × 53 points and a 400 mm^2^ area. The discrepancy principle is applied using η = 1.1 [29]. In the analysis, the correlation coefficient (CC) was computed and compared to the reference distribution after finishing the reconstruction process. The CC is expressed in Equations (7)–(9).

(7) $$M=\left(I-\frac{1}{N}\right)$$

(8) $$D=MM^{T}$$

(9) $$CC=\frac{x_{recon}^{T}MM^{T}x_{true}}{\sqrt{x_{recon}^{T}MM^{T}x_{recon}}\sqrt{x_{true}^{T}MM^{T}x_{true}}}=\frac{{\left(x_{recon},\;x_{true}\right)}_{D}}{\left|\left|x_{recon}|{|}_{D}\right|\right|x_{true}|{|}_{D}}$$

where 1 ∈ R^N^ is the all-one vector, and *I* is the identity matrix. The CC values indicate the correlation between ground truth $\left(x_{true}\right)\;$and reconstructed image $\left(x_{recon}\right)$ for different SNRs. The noise level was adjusted in the range of (5–30) with a 5 dB increment. The SNR is defined as the ratio of the maximum signal amplitude to the standard deviation of the noise. 

### 2.5. Measurement Scheme

In practical measurements, the magnitude of the excitation field is significantly larger than the nanoparticle response. The first harmonic response of particles, which is superimposed by the external excitation field, could not be extracted from a sensor signal. The third harmonic with the most prominent signal was chosen for the readout scheme. The sensor characteristics, including resonance frequency, sensitivity, noise density, and limit of detection, were measured and expressed in Table 1. As can be seen, the first resonance mode of the sensor was about 7.5 kHz. The excitation frequency was chosen to be tuned to 1/3 of the actual resonance frequency. The excitation field amplitude was set to 10 mT.

As shown in Figure 1, the measurement was conducted by moving the sample with respect to the fixed sensor position. The scanning area was set to 20 × 20 mm^2^, which includes 53 × 53 measurement points in an equidistant Cartesian grid. In order to position the sample, linear step motors and 2D XY-stage were utilized. The sensor-to-sample distance in the z direction was adjusted to 1 mm. The sensor output voltage was acquired in every measured position, and the fast Fourier transform (FFT) was computed based on the Blackman window function. The time length was set to 4096 samples at a 32 kHz sampling frequency, leading to a spectral resolution of 7.8 Hz. The complex peak signal at the resonance frequency, including the real and imaginary parts, was recorded and used in the reconstruction procedure.

## 3. Result and Discussion

The simulation results of image reconstruction for noiseless data and different SNR values are presented in this section. In addition, the experimental evaluation, which was performed based on the simulating prediction, is discussed subsequently.

### 3.1. Simulation Results

As shown in Figure 3a,b, the ground truth has been split into two single symbols and reconstructed reliably. Different colors, red for “B” and blue for “7”, indicate the phase difference for MNP types and lead to particle visual distinction. The final overall distribution, “B7”, shown in Figure 3c, could be achievable by combining related parts.

**Figure 4:**
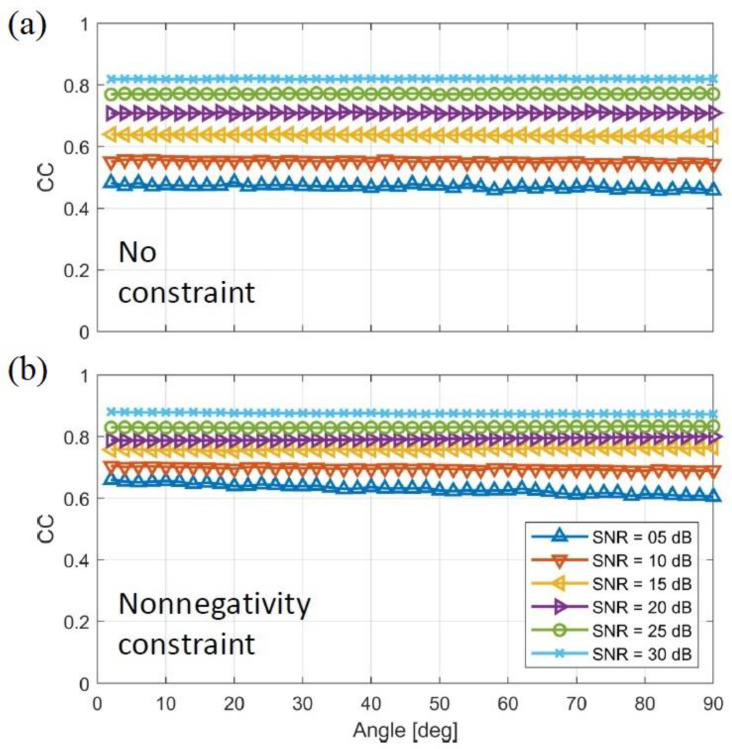
Simulation result for correlation coefficient evaluation on different particle phase responses, the x-axis denotes the phase difference between the simulated particle responses. (**a**) shows the correlation coefficient with no constraint applied and (**b**) with nonnegativity constraint.

As colored imaging for MPM is reliant on the different phase responses of the particles, the effect of particle phase difference on the correlation coefficient is illustrated in Figure 4a,b. In the first condition, the nonnegativity constraint was not considered in the reconstruction whose result shows the amplitude of the complex values (Figure 4a). The projection operator is applied for the second case and is depicted in Figure 4b. As it can be seen in both Figure 4a,b, increasing the SNR will increase the CC factor and spatial accuracy. The result was expected since the data are less contaminated by noise.

As shown in Figure 4a, the CC values did not change by increasing the particles’ phase difference. Similarly in Figure 4b, the CC values do not vary much with increasing particles’ phase difference. In direct comparison between Figure 4a,b, the overall correlation coefficient is higher when the nonnegativity constraint is applied, meaning that the incorporation of the constraint leads to a higher spatial accuracy of the reconstruction.

### 3.2. Experimental Results

**Figure 5:**
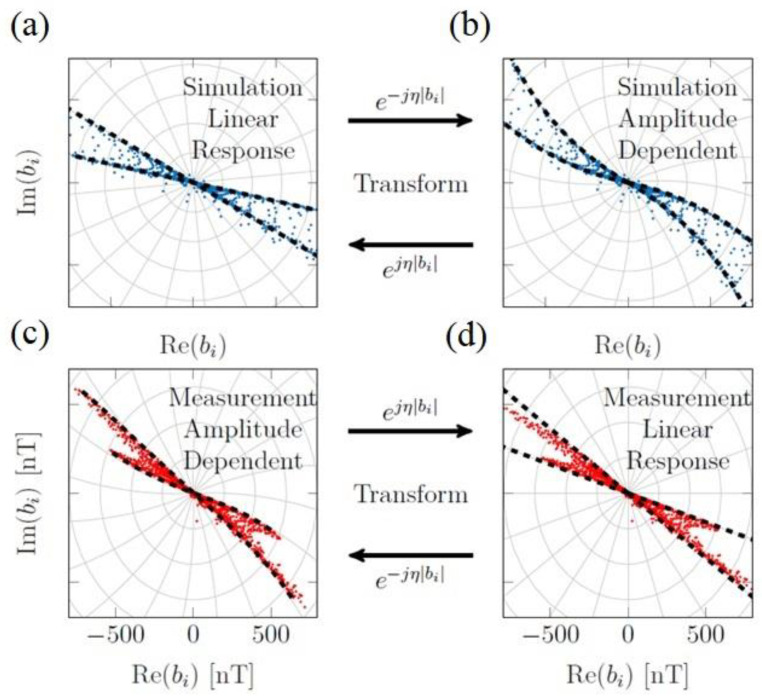
The real and imaginary parts of (**a**,**b**) simulated result and (**c**,**d**). Experimental measured magnetic field response for CT100 and ARA100 MNP types.

The experimental evaluation has been performed on the sample holder with a cylindrical shape, as illustrated in Figure S3 of Supplementary Data. In practical measurement, external conditions, such as noise and vibration, could couple with the sensor signal and subsequently form artifacts in the reconstructed image. Outlier points were manually removed from the data set before reconstruction. The MNP types were distinguished by acquiring the phase response near the corresponding particle region. The inevitable phase delay of about 90 degrees affected the original signal at the resonance frequency, which needs to be considered in the reconstruction. The experimental/simulation result for the real and imaginary parts of the magnetic field response of two magnetic nanoparticle types is shown in Figure 5a–d. The dashed line indicates the phase response of the nanoparticles. In the numerical simulation, most of the phase values of the measured signal lie between the particles’ phase responses, leading to a linear response. This trend can be seen clearly in Figure 5a. Despite this, both the sensor and MNP responses can affect the linearity of practical measurement. By affecting the magnetostrictive sensor layer with the magnetic field of MNPs, the working point of the sensor might be slightly shifted. Accordingly, phase and amplitude shifts could be expected in the measured voltage data. Figure 5b shows a simulated phase shift that depends on the field amplitude and can be transformed by a complex scalar $e^{-j\eta \left|b_{i}\right|}$, where $\left|b_{i}\right|$ is the voltage amplitude and η is a free parameter.

As can be seen in Figure 5a,b, the straight dashed lines are transformed into the curved form and indicate the amplitude-dependent phase. The measured signal for two MNP types is shown in Figure 5c,d. As indicated in Figure 5c, the phase values depend on the field amplitude to some extent. The independence of phase values is essential for the reconstruction procedure. To this end, the measured data were transformed to correct this effect. Figure 5d shows that after transforming, two straight lines can adequately enclose all of the measured points.

The change in sensor behavior during measurement could be described by the parametric resonator performance of the magnetoelectric sensor. More detail about the behavior of the utilized sensor can be found in the Supplementary Data (Figures S5–S7).

**Figure 6:**
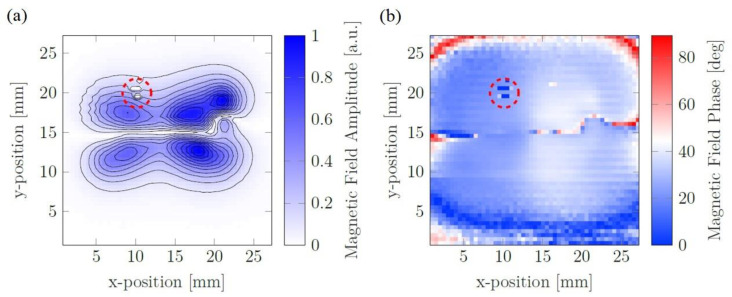
(**a**) Measured amplitude and (**b**) absolute value of the phase mod for the magnetic fields of the MNP distribution.

Figure 6 illustrates the measured voltage response of the sensor, which was split into a phase and an amplitude plot. The left and right regions of the plot, for both a and b, correspond to CT100 and ARA100 particle type, respectively. The outlier data have occurred in the measurement, indicated by the dashed circle, were manually removed before the reconstruction process. In order to have a better visualization, only the absolute value of the phase was depicted.

As shown in Figure 6b, the average phase for the CT100 is 23, which is 14 degrees higher than the ARA100 type. The dashed circles indicate the outliers that were coupled to the sensor signal.

The reconstruction process has been performed through the developed algorithm introduced in Section 2.2. The reconstructed image’s quality primarily depends on the accuracy and applicability of the forward model. In many cases, including magnetoelectric sensors, the direction of the sensor’s sensitivity axis is not precisely known and may vary during the measurement process due to variations in the bias field [28]. This source of uncertainty was considered in the inverse optimization algorithm. The estimated values for sensitivity axis and magnetic dipole moment are presented in Table 2. A hat symbol denotes the vector of unit length. It should be noted that both $\hat{s}$ and $\hat{m}$ vectors just indicate the directions of the mentioned quantities.

**Figure 7:**
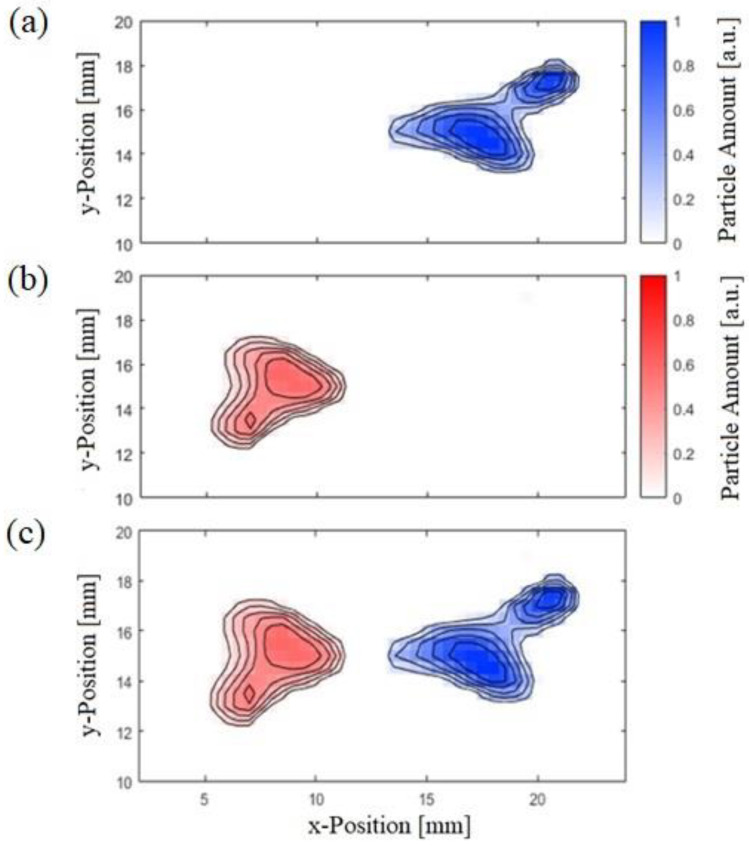
Reconstructed images for measured signal, (**a**) CT100 MNP and (**b**) ARA100 MNP type, (**c**) both MNP types simultaneously.

Figure 7 shows the reconstructed images for the raw measured data matrix present in Figure 6. As mentioned before, the CT100 and ARA100 MNP are represented in red and blue, respectively. For better visualization, both particle types are simultaneously plotted in Figure 7c.

As indicated in Figure 7, a clear spatial separation was observed between the two particle types, and hence, the shown approach could be considered for developing practical colored MPM systems. It must be emphasized that the proposed method can only distinguish two different particles. The reconstruction of three particles could be possible based on measuring the third and fifth harmonic simultaneously.

## 4. Conclusions

In this study, the capability of the magnetoelectric sensor for the colored MPM application was investigated via numerical simulation and experimental tests. The phase difference between MNPs responses was measured and utilized for distinguishing different nanoparticle types. The effect of the sensor sensitivity axis and dipole direction was considered in developing the forward model. Meanwhile, projected gradient descent was used as the optimization algorithm of the inverse problem and included a nonnegativity constraint for complex solution space. A numerical simulation was performed to predict the necessary conditions and reconstructed image results. The correlation coefficient between the ground-truth distribution and reconstruction is considered a performance metric. In the simulation of colored MPM, the outlined approach was implemented and tested against ground truths such that it could be used in the experimental investigation. The experimental investigation was carried out using the apparatus developed for two particle types, CT100 and ARA100. The particles were selected based on MPS evaluation and simulation results. The experimental data showed that the investigated particles had an average phase difference of 14 degrees, which was in line with the MPS results. Regarding the ME sensor feasibility for the colored MPM application, the high amplitude of excitation or particle response could affect the sensor properties and lead to generating the amplitude-dependent phase value. The phase values were adjusted using a complex exponential factor to compensate the amplitude dependence. As a result, the proposed technique can be considered a potential basis for the multi-colored image reconstruction process.

The various aspects, such as dynamic evaluation of magnetically labeled cells in complex 3D biomaterial geometry, multi-particle color imaging, and distinction of different particles in the same voxel, require further investigation, which can be pursued in future research.

## Figures and Tables

**Table 1:** Magnetoelectric sensor characteristics.

Characteristic (Symbol)	Unit	Value
Resonance frequency (f_r_)	Hz	7639
Sensitivity (S)	kV/T	93.0
Noise density (N_d_)	$\mathrm{nV}/\sqrt{\mathrm{Hz}}$	385
Limit of detection (LOD)	$\mathrm{pT}/\sqrt{\mathrm{Hz}}$	4

**Table 2:** The normalized values of sensor properties that were predicted by the optimization algorithm.

Spatial Component (Symbol)	$\hat{s}$	$\hat{m}$
X	0.0110	0.0588
Y	0.9958	0.0950
Z	0.0904	0.9937

## Data Availability

The data that support the findings of this study are available from the corresponding author upon request.

## Supplementary Materials

The following supporting information can be downloaded at: https://www.mdpi.com/article/10.3390/nano13020347/s1, Figure S1: The schematic illustration of MPM setup structure; Figure S2: Total harmonic spectrum of 150 µl CT100 particle suspension at 2.5 kHz excitation frequency in the range of [1–25] mT; Figure S3: Cylindrical sample holder with 4 mm thickness and separated containers; Figure S4: Depiction of a polyhedral cone in the complex plane for the x_i_ coefficients; Figure S5: The frequency response of the magnetoelectric sensor. The dashed line indicates the trend of the peak amplitudes for the different excitation signal; Figure S6: Parametric resonator behaviour of magnetoelectric sensor in MPI application; Figure S7: Multiple MPM measurements of the same sample measured in succession; Snippet S1. Projection procedure. References [30,31,32] are cited in the Supplementary Materials.
