# Supplementary material for: Numerical and Experimental Study of Colored Magnetic Particle Mapping via Magnetoelectric Sensors
Source: Nanomaterials (Basel). 2023 Jan 14;13(2):347. doi: 10.3390/nano13020347 (PMC9865076; doi:10.3390/nano13020347)
Supplement: Supplementary file 1 [file nanomaterials-13-00347-s001.zip › nanomaterials-2155661-supplementary.pdf]

nanomaterials

MDPI

Supplementary materials

Numerical and Experimental Study of Colored Magnetic Particle Mapping via Magnetoelectric Sensors

Ron-Marco Friedrich, Mohammad Sadeghi and Franz Faupel *

Chair for Multicomponent Materials, Institute of Materials Science, Kiel University, Kaiserstr. 2, D-24143 Kiel, Germany
* Correspondence: ff@tf.uni-kiel.de

Supplementary data

1. Magnetic imaging system apparatus

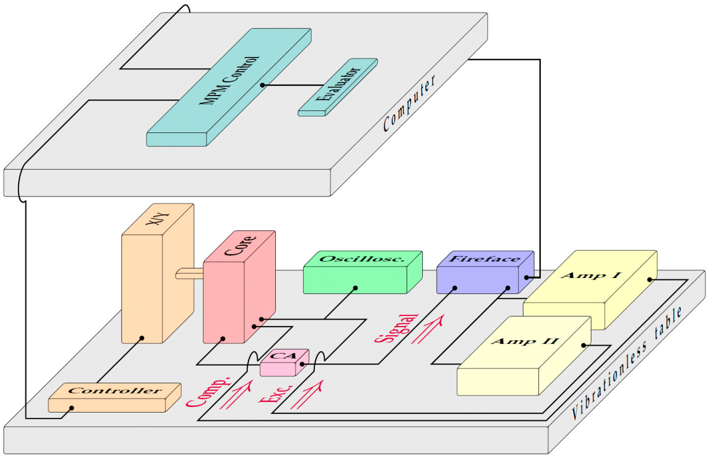

Figure S1. The schematic illustration of MPM setup structure.

2. Magnetic Particle Spectrometry

The effective magnetic moment of the particle's response for odd-numbered harmonic oscillations up to the 99th is illustrated in Figure S3. The third harmonic, which is of interest for the MPM system, is marked. Differently colored lines correspond to loops conducted with different maximum field strengths.

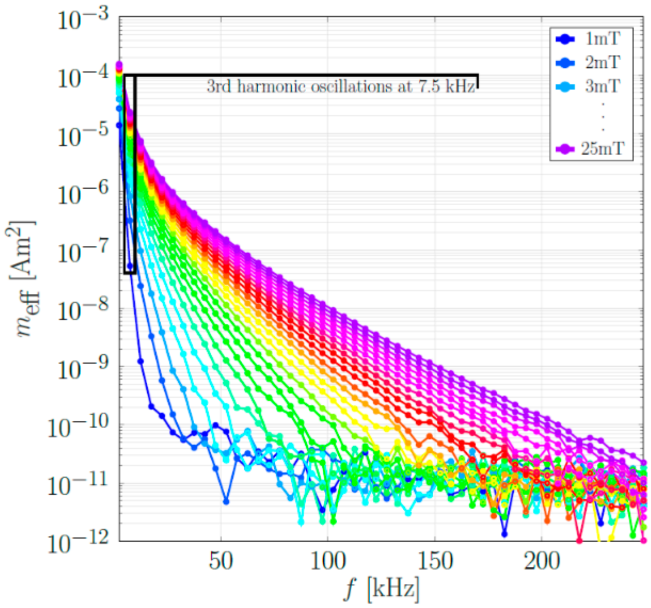

Figure S2. Total harmonic spectrum of 150 µl CT100 particle suspension at 2.5 kHz excitation frequency in the range of [1-25] mT.

3. Sample preparation

In this study, fluidMAG (chemicell-Berlin, Germany) nanoparticles have been used in the experimental test that is commercially designed for magnetic drug targeting, MRI diagnostics, and other bio-imaging applications. In this context, fluidMAG types with the same magnetite core and different sizes have been investigated. It should be mentioned that utilized MNPs can be used for different cell evaluation purposes, such as binding with cationic molecules, antibodies, and proteins [1].

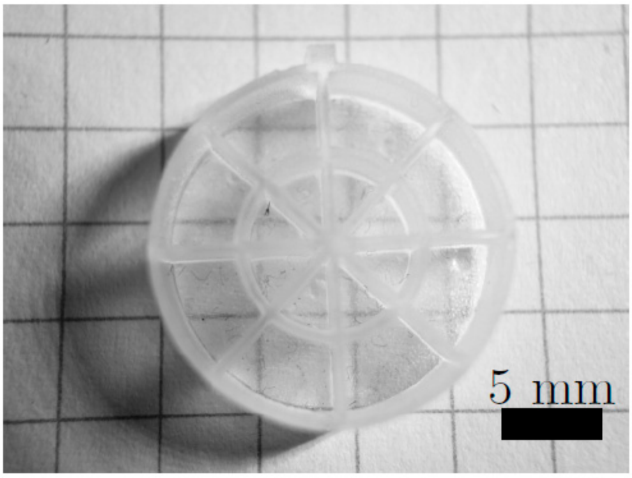

Figure S3. Cylindrical sample holder with 4 mm thickness and separated containers.

4. Nonnegativity constraint in complex plane

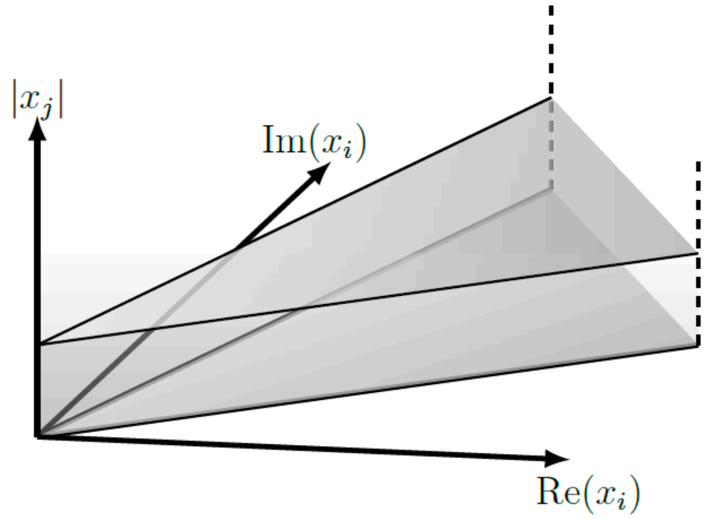

Figure S4. Depiction of a polyhedral cone in the complex plane for the x_i coefficients.

5. Projection Operator

For the projection, the following Matlab function was used:

Snippet S1. Projection procedure

```
function [x] = xConeProject(Q,x)
for k = 1:size(x,2)
    if sum(Q\x(:,k)<0)>0
        T = zeros(size(Q,2),1);
        for j = 1:size(Q,2)
            T(j,:) = sign(Q(:,j)'*x(:,k)).*(Q(:,j)'*x(:,k)).^2/norm(Q(:,j))^2;
        end
        v = Q(:,T==max(T));
        x(:,k) = max(v'*x(:,k)/(v'*v),0)*v;
    end
end
end
```

6. Magnetoelectric Sensor behaviour

The ME sensor acts as a mechanical resonator and converts the magnetic fields into a voltage signal. The oscillation amplitude of the sensor will increase significantly at the mechanical resonance, which leads to high sensitivity at the resonance frequency. In this context, under applying a very high magnetic excitation amplitude, a nonlinearity effect will appear. Hence, hardening/softening will tilt the frequency response and the sensor characteristics detune, consequently. The effect of excitation amplitude on the stress softening/hardening for the utilized sensor was measured in the MPI-related range. As illustrated in Fig. S5, the frequency response of the sensor first shifts to lower values (softening) followed by a shift to higher frequencies (hardening). The maximum achievable amplitude will decrease at a higher excitation signal.

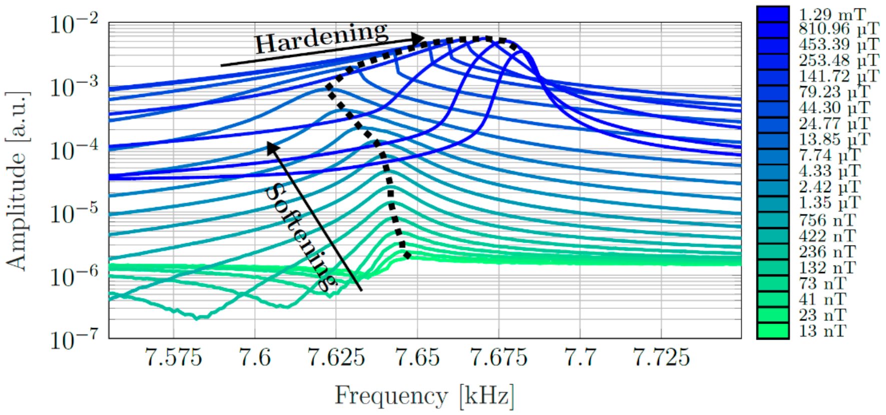

Figure S5. The frequency response of the magnetoelectric sensor. The dashed line indicates the trend of the peak amplitudes for the different excitation signal.

Figure S6 shows the frequency response of the utilized ME sensor for strong magnetic excitation. The parametric resonator occurs at frequencies of $\frac{2f_1}{n}$, where f_1 is the resonance frequency and n is a positive integer. Additional resonance peaks have occurred in parametric resonances for the second bending mode in $\frac{f_2}{n}$. A detuning effect could be caused by both the excitation field and the MNPs signal during MPI measurement. Accordingly, the sensor characteristics change periodically, and this can be viewed as a time-varying sensitivity. In other words, a higher harmonic excitation field could be generated due to the sensor itself and interfere with the MNP signal, which subsequently inhibits the proper measurement of the nanoparticle signal. The parametric sensor behavior and experimental results align with previous reports [2,3].

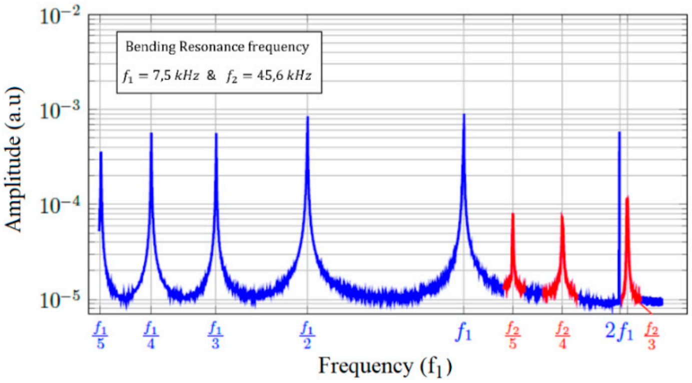

Figure S6. parametric resonator behavior of magnetoelectric sensor in MPI application.

For magnetic particle mapping, the stability and reproducibility of the sensor response should be taken into account. In this regard, several MPM measurements of the same sample have been performed in succession. The measured signal was converted to nT via the ME coefficient. As can be seen in Figure S7, the amplitude of the measured signal for all iterations is in the same range.

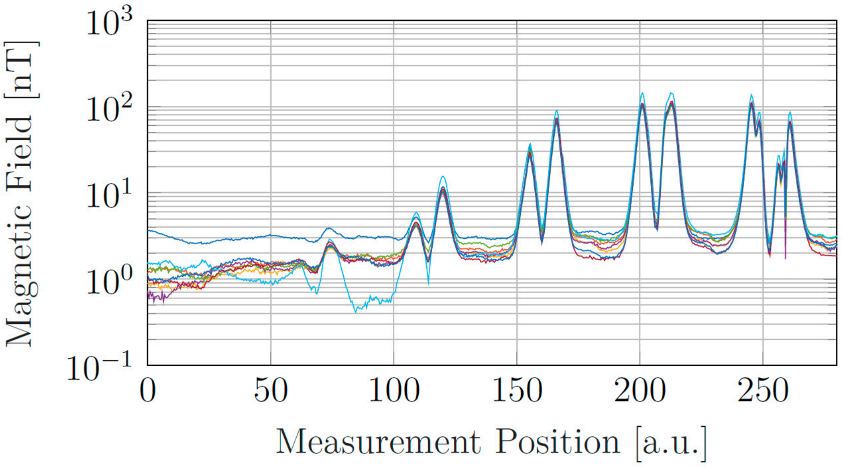

Figure S7. Multiple MPM measurements of the same sample measured in succession.

References

1. http://www.chemicell.com/products/Magnetic_Nanoparticle/Magnetic_Nanoparticles.html (accessed on 9 January 2023).

2. Fetisov, L.Y.; A Burdin, D.; A Ekonomov, N.; Chashin, D.V.; Zhang, J.; Srinivasan, G.; Fetisov, Y.K. Nonlinear magnetoelectric effects at high magnetic field amplitudes in composite multiferroics. *J. Phys. D Appl. Phys.* **2018**, *51*, 154003.

3. Jia, Y.; Yan, J.; Soga, K.; Seshia, A.A. Parametric resonance for vibration energy harvesting with design techniques to passively reduce the initiation threshold amplitude. *Smart Mater. Struct.* **2014**, *23*, 065011.
